# Book Reviews

**Published:** 1898-10

**Authors:** 


### Book Reviews.

---

GLAUCOMA, its Symptoms, Varieties, Pathology and Treatment. By Alex. W. Sterling, M.D., C.M. (Edin.), D.P.H. (Lond.), Atlanta, Ga.

This book comprises in great part a series of lectures delivered to the students of the "Post-Graduate Medical School and Hospital," of New York, and published serially in the "Annals of Ophthalmology," and gives evidence of most careful research, and a thorough knowledge of the subject on the part of the author.

---

### BOOKS RECEIVED.

Twelfth Century Practice of Medicine.
Vol. XV.—Infectious Diseases. Wm. Wood & Co.
Simon's Chemistry. Lee Bros.
Osler's Practice. D. Appleton.
The Mind Reader. L. M. Phillips, F. Tennyson Neely.
Diet and Food, Haig. P. Blakeston.
Diet for the Sick. Illustrated Medical Journal Co.

